# Peer feedback and Chinese medical students’ English academic writing development: a longitudinal intervention study

**DOI:** 10.1186/s12909-023-04574-w

**Published:** 2023-08-16

**Authors:** Chenze Wu, Yao-Wu Zhang, Albert W. Li

**Affiliations:** 1 Moray House School of Education and Sport, University of Edinburgh, Edinburgh, UK; 2 School of Medicine, Johns Hopkins University, Baltimore, USA; 3 School of Education, University of California, Irvine, USA

**Keywords:** Peer feedback, Academic writing, Chinese medical students, Writing instruction

## Abstract

**Background:**

Studies have documented that utilizing peer feedback can enhance students’ English academic writing skills. Little is known, however, about the effects of incorporating peer feedback to enhance English as a second language (L2) medical students’ academic writing performance.

**Methods:**

This longitudinal interventional study examines Chinese medical students’ English academic writing skills development via peer feedback in four parallel classes over an 18-week semester between the experimental and control groups (*n* = 124).

**Results:**

Significant increases in the experimental group’s performance in the post-test were found after 18-week instructions (pre- vs. post-test: overall score, *p* < .001; task response, *p* < .001; coherence and cohesion, *p* < .001; lexical resource, *p* < .001; grammatical range and accuracy, *p* < .001), and the effects were retained in the delayed post-test 6 weeks later (post- vs. delayed post-test: overall score, *p* = .561; task response, *p* = .585; coherence and cohesion, *p* = .533; lexical resource, *p* = .796; grammatical range and accuracy, *p* = .670). Little improvement was found in the control group in the post-test (pre- vs. post-test: overall score, *p* = .213; task response, *p* = .275; coherence and cohesion, *p* = .383; lexical resource, *p* = .367; grammatical range and accuracy, *p* = .180) or the delayed post-test (post- vs. delayed post-test: overall score, *p* = .835; task response, *p* = .742; coherence and cohesion, *p* = .901; lexical resource, *p* = .897; grammatical range and accuracy, *p* = .695). Between-group comparisons indicate that the experimental group outperformed the control group in the post- and the delayed post-tests, as shown in their overall score and scores on the four components.

**Conclusions:**

Incorporating peer feedback into process-oriented medical English writing classroom teaching can effectively enhance Chinese medical students’ English academic writing skills over time, while the traditional product-oriented writing instructions had little help in improving Chinese medical students’ academic writing skills. This longitudinal intervention study develops our understanding of the effectiveness of peer feedback in L2 academic writing pedagogy. It offers instructional implications for L2 writing teachers to teach English academic writing among medical students in China and beyond. Limitations and suggestions for future studies are discussed.

## Introduction

Researchers have proposed several approaches for teaching writing, e.g., the product- and process-oriented approaches [13, 39]. The product-oriented approach emphasizes the final piece of writing, focusing on errors in writing, fluency, and grammatical structure [5, 29]. In other words, it emphasized “what students produced” instead of “how to produce.” Further, previous studies documented that writing teachers sometimes cannot realize the characteristics and differences of each student while applying a product-oriented approach [6], which may cause students’ negative attitudes toward writing learning [43]. On the contrary, the process writing approach views students as the center of teaching activities, and teachers are positive organizers and participants [20]. Moreover, Mehr’s [22] study has shown that the process writing approach could cultivate students’ positive attitudes toward English writing and significantly enhance learners’ English writing performance. Considering these benefits, the process writing approach has been utilized in various writing teaching contexts. Specifically, during this student-centered learning process, a peer-supported positive learning environment can be constructed, which stresses students’ values in academic tasks [17, 18, 47].

Peer feedback, a commonly used peer-supported positive learning strategy in the writing process, refers to a process where learners provide feedback on their peers’ work in oral or written forms and learners also receive feedback from other peers (see [1, 18, 20, 27, 40, 44, 45, 47]). The objective of giving peer feedback is to enable students to understand the limitations of their work and offer corresponding suggestions to help them improve their current work [2, 3, 19, 25, 27]. Furthermore, using peer feedback in writing learning could provide students with opportunities to learn from others since they need to critically evaluate peers’ work and identify both benefits and shortcomings before generating feedback [12, 14, 15, 24, 38]. Compared with traditional written corrective feedback from writing teachers, peer feedback could help learners receive feedback more quickly and may include more informative details for writing improvement [17, 34, 35].

Given the advantages of peer feedback above and the potential of utilizing peer feedback for English writing instructions, previous studies have incorporated peer feedback activities into the process-oriented approach for improving students’ writing performance (e.g., [20, 40, 42, 45]). During the process of giving and receiving peer feedback, students can exchange their thoughts with other peers and reflect on their drafts at the same time. By thinking from other readers’ points of view and analyzing expectations from audiences, students can obtain “reader awareness”, which might help improve their writing competence [10, 20]. Previous empirical studies have also shown that peer feedback could significantly improve learners’ English writing skills in different learning contexts (e.g., [1, 27, 36, 37, 46, 47]). For example, a study with a quasi-experimental design explored the effects of peer feedback on the English writing performance of English major students (*n* = 198) in a Chinese university [46]. They demonstrated that, in comparison with traditional written corrective feedback from writing teachers, peer feedback could significantly enhance English major students’ writing performance in China. Similarly, Uymaz [36] investigated the effects of peer feedback on Turkish university students’ L2 English writing development and revealed that students’ writing performance was significantly improved after receiving peer feedback on their four English writing tasks. However, as argued by educational researchers, applying peer feedback to enhance non-native medical students’ English writing skills over time is still underexplored [7]. Additionally, the majority of the current intervention studies only focused on the change in students’ writing skills between the pre-test and the post-test (e.g., [36, 46]), while delayed post-test should be conducted to scrutinize whether the effects of certain instructions can be sustained after the interventions were completed [41].

To address some of the existing research gaps, the current study applies a longitudinal intervention design to investigate the effects of peer feedback on L2 medical students’ academic writing skills development over time. Specifically, this study is guided by the following two research questions (RQ):

- **RQ1**: Does the use of peer feedback improve Chinese medical students’ English academic writing development over time?
- **RQ2**: Is there any difference in the effects of peer feedback and traditional writing instructions on Chinese medical students’ English academic writing development?

## Methods

### Participants

A total of 124 freshmen medical students participated in the study at an eastern-Chinese university. After the participants provided their written informed consent to participate in this study, they were randomly separated into the experimental group (EG, *n* = 62) and the control group (CG = 62). Students in the experimental group were 10 males and 52 females between 18 and 19 (M = 18.79, SD = 0.410). Similarly, students in the control group were 9 males and 53 females between 18 and 19 (M = 18.87, SD = 0.338). Regarding their past English learning experience, they started to learn English in the first year of primary school. In addition, the homogeneity tests were run to ensure students’ writing abilities were homogenous in the pre-test. The results illustrated that students’ writing skills in overall scores and the five subscores were similar at the pre-test (see Results section).

### Writing classes

The current study was conducted within an 18-week English writing course, which aims to enhance medical students’ English writing performance in a wide range of genres. The writing teachers in both the experimental group and the control group have obtained their Ph.D. (Doctor of Philosophy) degrees in English language education and have taught English writing for 15 years. Four key genres (argumentative writing, narrative writing, descriptive writing, and literature review writing) were chosen as the teaching target for both groups. Although the English writing tasks involved various topics such as education, career, and culture, most of the writing topics were health-related due to their medical major.

### Research design

Peer feedback activities were implemented in the experimental group and the writing course design. The writing teachers for the experimental group (2 parallel classes) incorporated peer feedback activities, while the teachers for the control group (2 parallel classes) applied the conventional product-oriented approach.

In the experimental group, students received feedback on different writing dimensions from peers, and eventually completed a revised draft each week. To help students provide effective peer feedback, the Department of English of the university arranged weekly peer feedback workshops to teach students how to use a rubric to offer feedback on each dimension of their peers’ work. Specifically, the details of each dimension on the rubrics were explained in the weekly workshops. Writing samples for different score ranges were also presented, and teachers explained how to use the rubric to provide feedback to these writing samples, which could ensure the inter-rater reliability and the uniformity of the marking process to a large extent. Furthermore, when they experienced difficulties in giving feedback to their peers, they could immediately raise their hands and seek help from the teacher in writing classes. The rubric used in this study was adapted from the rubric for marking writing sections of College English Test-6 (CET-6) [4], which is a commonly used and taught rubric in college English courses in China. The adapted rubric is a 9-point scale, including four dimensions: task response, coherence and cohesion, lexical resource, and grammatical range and accuracy. The overall writing scores were the mean of these four dimensions. Correspondingly, the conventional product-oriented approach was implemented to teach writing in the control group, i.e., the writing teacher emphasized grammar accuracy, vocabulary breadth, and the model texts in the coursebook. Students only need to write one draft and teachers would give some feedback, focusing on the lexical and grammatical aspects. After that, students directly corrected these mistakes on the originals.

To alleviate external factors affecting learners’ writing performance, weekly pre-class teaching training sessions were organized with the two teachers for the experimental group to guide them to implement the same teaching procedures, and the two teachers for the control group attended other sessions for the same purpose. The first author observed the actual writing classes to ensure that teachers teach English academic writing in a planned manner.

### Pre-, post-, and delayed post-tests

At the pre-test stage, a mock writing test for CET-6 was arranged by the Department of English at this university, and students should finish the writing section in the classroom. The writing samples were collected to obtain baseline data from the experimental and control groups. Then, 18-week writing instructions were carried out in the experimental group. At the post-test stage, another mock writing test was conducted again immediately after the interventions. In addition, to investigate the follow-up effects, we conducted a delayed post-test 6 weeks after the intervention was completed. The topics of the mock writing test for pre-, post-, and delayed post-tests were adapted from the writing section of the CET-6 test, which requires students to write an essay with around 200 words within 30 minutes. Three experienced English writing teachers were consulted to ensure the comparability and feasibility of the two writing topics. And then, students from another parallel class were invited to finish the two tasks to guarantee that the difficulty of the two topics was moderate for freshmen medical students.

### Scoring

A double-blinded expert (two teachers) assessment mechanism was applied to score students’ writings. The 9-point scoring rubric was used to rate all essays from four dimensions with equal weight: task response, coherence and cohesion, lexical resource, and grammatical range and accuracy. Two teachers were invited to an academic seminar to guarantee acceptable inter-rater reliability and the uniformity of marking criteria. At first, twenty compositions were given to the two writing teachers, and they scored the compositions individually. Then, they went to check whether they could give the same mark on the same compositions. If not, they were asked to have a communication to explain why they scored this mark. During this process, they can agree on the scoring standard, and then they were allowed to evaluate the rest of the compositions independently. Finally, Cronbach’s Alpha was calculated and reported at 0.802 in pre-tests, 0.855 in post-tests, and 0.875 in delayed post-tests, which indicated their scoring has good reliability.

### Statistical analyses

The assumptions of the statistical tests were checked via standard diagnostic tests and procedures. The visual inspection of histograms, normal Q-Q, normal P-P, box plots, skewness and kurtosis, and the Shapiro-Wilk tests were used to check the normality of distribution, Levene’s test was used for homogeneity of variance, and Mauchily’s test was used for sphericity of the data. Then, 2 × 3 mixed factorials analysis of variance (ANOVAs) and Friedman test were conducted to investigate the effects of peer feedback on L2 students’ writing skills. Besides, simple effect analysis and Wilcoxon signed-ranks tests were used as the posthoc test within groups. Where there were multiple comparisons, Bonferroni corrections were used to avoid Type I errors. Partial eta squared (*η*_*ρ*_^*2*^) was used for measuring the effect sizes for mixed ANOVAs. Cohen’*d* was calculated for the t-tests, and r was calculated for Wilcoxon and Mann–Whitney tests. The interpretation of the results was based on Cohen’s (1992) classification that *η*_*ρ*_^*2*^ values of 0.01, 0.06, 0.14; *d* values of 0.20, 0.50, and 0.80; and *r* values of 0.10, 0.30, and 0.50 were considered small, medium, and large, respectively.

## Results

**Fig. 1:**
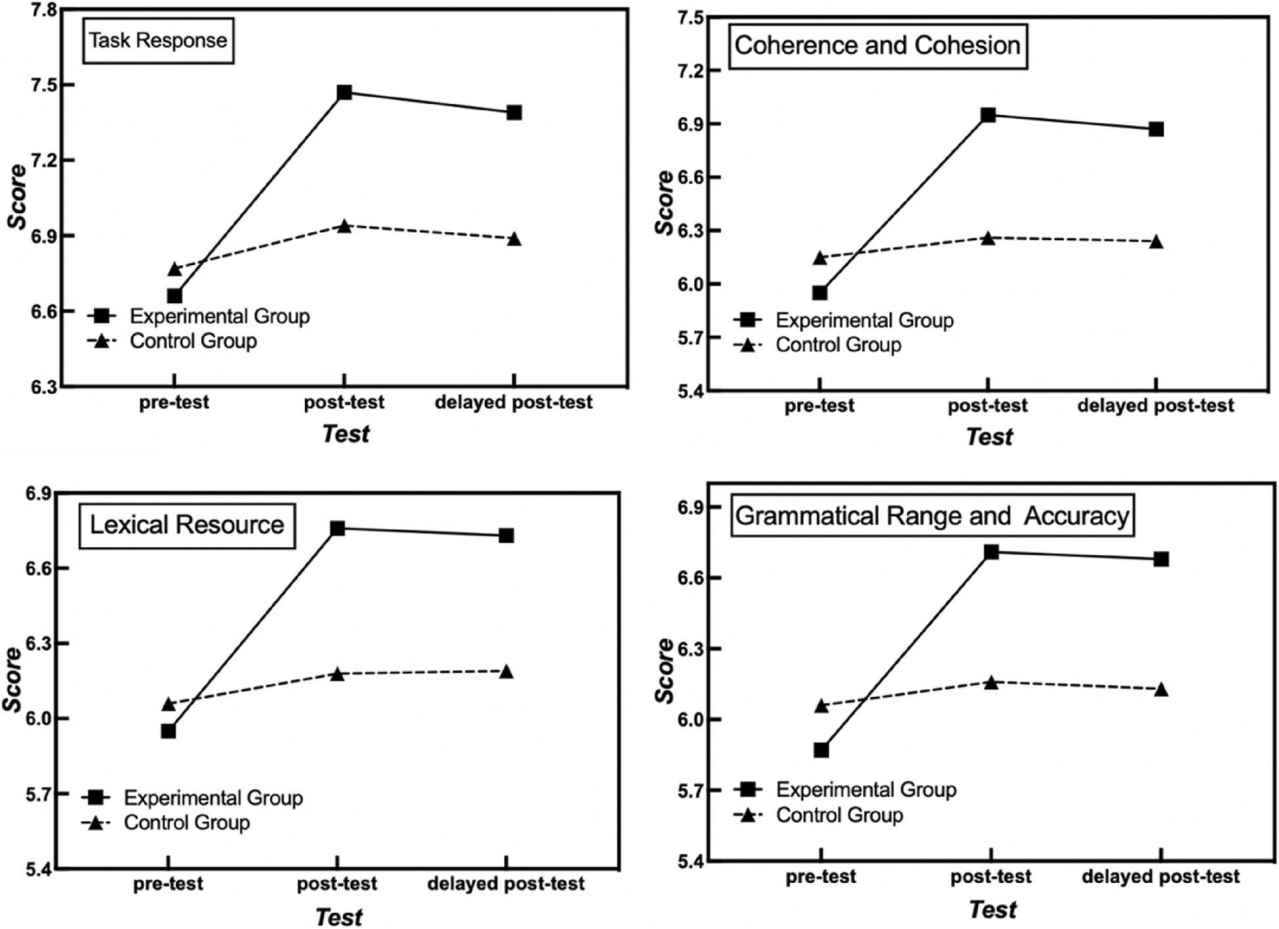
Group means across time for subscores

**Fig. 2:**
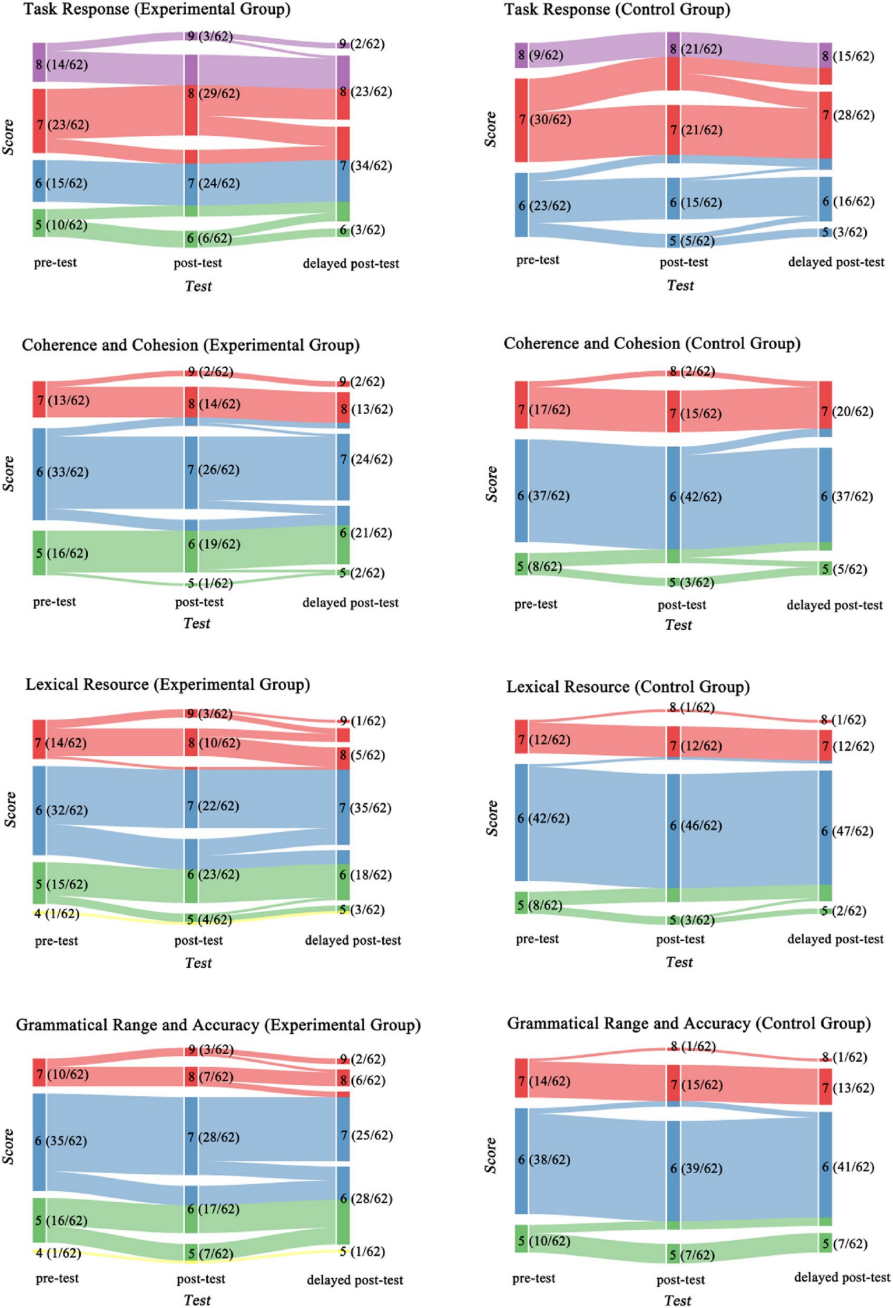
Individual means across time for subscores

Descriptive statistics of students’ overall writing scores and subscores for both the experimental and control groups in the pre-, post-, and delayed post-tests are shown in Table 1. The line charts in Fig. 1 visually present the changes in group means across time for subscores, and the alluvial plots in Fig. 2 present the changes in individual means across time for subscores.

**Table 1:** Means and standard deviations of the overall score and subscores across time in the experimental and control groups

**Component**	**Group**	**Pre-Test**		**Post-Test**		**Delayed Post-Test**	
		**Mean**	***SD***	**Mean**	***SD***	**Mean**	***SD***
**Overall**	EG	6.11	0.55	6.97	0.71	6.92	0.62
	CG	6.26	0.45	6.38	0.41	6.36	0.43
**Task Response**	EG	6.66	1.00	7.47	0.74	7.39	0.64
	CG	6.77	0.69	6.94	0.96	6.89	0.83
**Coherence and Cohesion**	EG	5.95	0.69	6.95	0.86	6.87	0.90
	CG	6.15	0.62	6.26	0.60	6.24	0.59
**Lexical Resource**	EG	5.95	0.73	6.76	0.97	6.73	0.75
	CG	6.06	0.57	6.18	0.53	6.19	0.51
**Grammatical Range and Accuracy**	EG	5.87	0.69	6.71	0.98	6.68	0.81
	CG	6.06	0.62	6.16	0.63	6.13	0.61

*EG* Experimental group, *CG* Control group, *SD* Standard deviation

Given that the assumptions of normal distribution, homogeneity, and sphericity were met, mixed ANOVAs were conducted for the overall writing scores and subscores including task response, coherence and cohesion, and lexical resource. Friedman test was conducted as an alternative analysis for grammatical range and accuracy scores since the scores were not normally distributed. The results of the baseline test illustrate that students’ writing skills in overall scores and the five subscores were similar at the pre-test (overall, *p* = 0.115; task response, *p* = 0.444; coherence and cohesion, *p* = 0.135; lexical resource, *p* = . 367; grammatical range and accuracy, *p* = . 117).

### Effect on overall writing score over time

As shown in Table 2, mixed-design ANOVA results indicated that there was a significant main effect of time, *F* (2, 369) = 31.85, *p* < 0.001, *η*_*ρ*_^*2*^ = 0.15, a significant main effect of group, *F* (1, 60) = 34.60, *p* < 0.001, *η*_*ρ*_^*2*^ = 0.09, and a significant interaction effect for time × group, *F* (5, 366) = 18.61, *p* < 0.001, *η*_*ρ*_^*2*^ = 0.09, on students’ overall writing scores.

**Table 2:** Summary of 2 (group) × 3 (time) mixed-design analysis of variance

**Effect**	***F***	***df***	***P***	***η***_***ρ***_^***2***^
**Overall**				
***Time***	31.85	(2, 369)	0.000**	0.15
***Group***	34.60	(1, 60)	0.000**	0.09
***Time* × *Group***	18.61	(5, 366)	0.000**	0.09
**Task Response**				
***Time***	12.69	(2, 369)	0.000**	0.07
***Group***	12.95	(1, 60)	0.000**	0.03
***Time* × *Group***	6.08	(5, 366)	0.003*	0.03
**Coherence and Cohesion**				
***Time***	22.74	(2, 369)	0.000**	0.11
***Group***	25.43	(1, 60)	0.000**	0.07
***Time* × *Group***	14.64	(5, 366)	0.000**	0.07
**Lexical Resource**				
***Time***	17.75	(2, 369)	0.000**	0.09
***Group***	21.36	(1, 60)	0.000**	0.06
***Time* × *Group***	9.61	(5, 366)	0.000**	0.05

^*^p < .05; **p < .001

Then, a simple effect analysis was used to explain the time × group interaction effect further. Within-subject comparisons (see Table 3) revealed that peer feedback can help students in the experimental group to develop their overall writing scores over time (pre- vs. post-test, *p* < 0.001; pre- vs. delayed post-test, *p* < 0.001), and the effect was retained in the delayed post-test (post- vs. delayed post-test, *p* = 0.561). Nevertheless, the overall writing score of students in the comparison group did not improve significantly across the three tests (pre- vs. post-test, *p* = 0.213; post- vs. delayed post-test, *p* = 0.835).

**Table 3:** Simple effect analysis: within-subject comparisons

**Subjects**	**Pre- vs. Post-Test**				**Post- vs. Delayed Post-Test**				**Pre- vs. Delayed Post-Test**			
	**EG**		**CG**		**EG**		**CG**		**EG**		**CG**	
	***SE***	***p***	***SE***	***p***	***SE***	***p***	***SE***	***p***	***SE***	***p***	***SE***	***p***
**Overall**	0.10	0.000**	0.10	0.213	0.10	0.561	0.097	0.835	0.10	0.000**	0.10	0.299
**Task Response**	0.15	0.000**	0.15	0.275	0.15	0.585	0.15	0.742	0.15	0.000**	0.15	0.444
**Coherence and Cohesion**	0.13	0.000**	0.13	0.383	0.13	0.533	0.13	0.901	0.13	0.000**	0.13	0.455
**Lexical Resource**	0.13	0.000**	0.13	0.367	0.13	0.796	0.13	0.897	0.13	0.000**	0.13	0.302

*EG* Experimental group, *CG* Control group, *SD* Standard deviation

^**^p < .001

Further, between-group comparisons (see Table 4) suggested that the overall writing performances of the two groups were similar in the pre-test (*p* = 0.115), while the experimental group significantly outperformed the comparison group in the post-test, *p* < 0.001, with large effect size, *d* = 1.02, and in the delayed post-test, *p* < 0.001, with large effect size, *d* = 1.04.

**Table 4:** Simple effect analysis: between-subject comparisons

**Subjects**	**Pre-test**		**Post-test**		**Delayed post-test**	
	***p***	**Cohen’s d**	***p***	**Cohen’s d**	***p***	**Cohen’s d**
**Overall**	0.115	-0.30	0.000**	1.02	0.000**	1.04
**Task Response**	0.444	-0.13	0.000**	0.62	0.001*	0.68
**Coherence and Cohesion**	0.135	-0.30	0.000**	0.93	0.000**	0.83
**Lexical Resource**	0.367	-0.17	0.000**	0.74	0.000**	0.84

^*^p < .05; **p < .001

### Effect on individual writing dimensions over time

The results of 2 × 3 mixed factorials analysis of variance (ANOVAs) suggested that there was a significant main effect of time, *F* (2, 369) = 12.69, *p* < 0.001, *η*_*ρ*_^*2*^ = 0.07, a significant main effect of group, *F* (1, 60) = 12.95, *p* < 0.001, *η*_*ρ*_^*2*^ = 0.03, and a significant interactive effect of time × group, *F* (5, 366) = 6.08, *p* = 0.003, *η*_*ρ*_^*2*^ = 0.03, with respect to the task response score. Furthermore, a significant main effect of time, *F* (2, 369) = 22.74, *p* < 0.001, *η*_*ρ*_^*2*^ = 0.11, a significant main effect of group, *F* (1, 60) = 25.43, *p* < 0.001, *η*_*ρ*_^*2*^ = 0.07, and a significant interactive effect of time × group, *F*(5, 366) = 14.64, *p* < 0.001, *η*_*ρ*_^*2*^ = 0.07 were found in terms of the coherence and cohesion score. The mixed-design ANOVA results also showed that there was a significant main effect of time on lexical resource, *F* (2, 369) = 17.75, *p* < 0.001, *η*_*ρ*_^*2*^ = 0.09, a significant main effect of group, *F*(1, 60) = 21.36, *p* < 0.001, *η*_*ρ*_^*2*^ = 0.06, and a significant interactive effect of time × group, *F*(5, 366) = 9.61, *p* < 0.001, *η*_*ρ*_^*2*^ = 0.05.

As shown in Table 3, simple effect analysis further explained the improvement in task response scores over time for students in the experimental group (pre- vs. post-test, *p* < 0.001; pre- vs. delayed post-test, *p* < 0.001), and the effect was maintained in the delayed post-test (post-test vs. delayed post-test, *p* = 0.585), whereas the score for the control group did not improve over time (pre- vs. post-test, *p* = 0.275; post- vs. delayed post-test, *p* = 0.742). Moreover, a simple effect analysis revealed that peer feedback enhanced the coherence and cohesion scores for students in the intervention group, and the effect was retained in the delayed post-test (pre- vs. post-test, *p* < 0.001; pre- vs. delayed post-test, *p* < 0.001; post-test vs. delayed post-test, *p* = 0.533). However, no significant improvement was found for the comparison group over the three-time points (pre- vs. post-test, *p* = 0.383; post- vs. delayed post-test, *p* = 0.901). Simple effect analysis also shows that the lexical resource scores improved significantly for the experimental group (pre- vs. post-test, *p* < 0.001; pre- vs. delayed post-test, *p* < 0.00), and the effect was retained in the delayed post-test (post- vs. delayed post-test, *p* = 0.796). However, for the control group, no significant improvement was found over time (pre- vs. post-test, *p* = 0.367; post- vs. delayed post-test, *p* = 0.897).

Additionally, the task response scores of the two groups in the pre-test showed no significant difference (*p* = 0.444), while students in the experimental group performed much better than those in the control group in the post-test (*p* < 0.001) and in the delayed post-test (*p* < 0.001), with medium effect sizes, d = 0.62 and d = 0.68, respectively. Between-group comparisons (see Table 4) revealed that no significant difference was found between the two groups in coherence and cohesion scores in the pre-test (*p* = 0.135). However, the experimental group showed better performance regarding coherence and cohesion than the control group in the post-test, *p* < 0.001, with a large effect size (d = 0.93), and in the delayed post-test, *p* < 0.001, with a large effect size (d = 0.83). Between-group comparisons revealed that no significant difference was found between the two groups in lexical resource scores in the pre-test (*p* = 0.367). However, the experimental group showed better performance regarding lexical resource scores than the control group in the post-test, *p* < 0.001, with a medium effect size d = 0.74), and in the delayed post-test, *p* < 0.001, with a large effect size, d = 0.84.

Given that the scores were not normally distributed, the Friedman test examined the differences in grammatical range and accuracy scores over the three tests. Table 5 shows that peer feedback had effects on the grammatical range and accuracy scores for the experimental group (*χ*^*2*^ = 43.33, *p* < 0.001), and had no effects on that of the control group across time (*χ*^*2*^ = 1.24, *p* = 0.537).

**Table 5:** Friedman test results for the EG and CG on grammatical range and accuracy

	***N***	***χ***^***2***^	***df***	***P***
**EG**	62	43.33	2	0.000**
**CG**	62	1.24	3	0.537

*EG* Experimental group, *CG* control group, *SD* Standard deviation

^**^p < .001

Post-hoc analysis with Wilcoxon signed-rank test was conducted with a Bonferroni correction applied, resulting in a significant level set as *p* < 0.017 (0.05/3≈0.017). There was a statistically significant improvement in the grammatical range and accuracy scores in the experimental group in the pre-test vs. the post-test (*z* = -4.84, *p* < 0.001, *r* = -0.44), the pre-test vs. the delayed post-test (*z* = -4.90, *p* < 0.001, *r* = -0.44). And no significant difference was found for the experimental group in the post-test vs. the delayed post-test (*z* = -0.43, *p* = 0.670, *r* = -0.04), meaning that the effect was retained in the delayed post-test. However, no significant difference was found in the grammatical range and accuracy scores in the control group in the pre-test vs. the post-test (*z* = -1.34, *p* = 0.180, *r* = -0.12), the pre-test vs. the delayed post-test (*z* = -0.71, *p* = 0.480, *r* = -0.06), and the post-test vs. the delayed post-test (*z* = -0.39, *p* = 0.695, *r* = -0.04), indicating that the traditional product-oriented approach had little effects on students’ grammatical range and accuracy scores. Additionally, Mann-Whitney U tests were conducted to compare the differences between the two groups at the three tests. The results showed that students in the experimental group and the control group in the pre-test were quite similar concerning the grammatical range and accuracy scores (*U* = 1645.00, *p* = 0.117, *r* = 0.20), whereas statistically significant differences were found in the grammatical range and accuracy scores between the experimental group and the control group in the post-test (*U* = 1254.50, *p* < 0.001, *r* = 0.45) and the delayed post-test (*U* = 1215.00, *p* < 0.001, *r* = 0.49).

## Discussion and conclusion

The current study aimed to investigate the effects of peer feedback on enhancing medical students’ English writing skills development over time at a university in China. Peer feedback (see Table 6) was used to design the teaching instructions for the experimental group in an 18-week semester, while the traditional writing teaching approach was applied to the control group. The overall writing score and score in each dimension (*task response*, *coherence and cohesion*, *lexical resource*, and *grammatical range and accuracy*) for the experimental group and the control group were compared at both pre- and post-test stages.

**Table 6:** Illustrative examples of revisions based on peer feedback in the second draft

**Ss**	**Peer Feedback Received**	**Revision(s)**
**Lisa**	*Coherence and Cohesion* - I think the topic sentence in the second paragraph of your essay can be more informative by adding reasons in this sentence before you give detailed explanation below.	It is evidently reasonable for me to believe that learning traditional plays and works of theaters has many advantages *due to the fact that this would exert positive effects on cultural maintenance*.
**Mike**	*Lexical Resource* - I suggest you can replace “wasting time” with the adjective “time-consuming” to make your essay more academic.	It is exceedingly necessary to point out that the process of learning traditional plays and theaters is *time-consuming*.
**Tina**	*Grammatical Range and Accuracy* - I consider the “have” in the first sentence of the last paragraph should be changed into “has” because it follows after “it”. Besides, I believe you should pay more attention to the usage of the third person singular.	We may safely reach the conclusion that it *has* both benefits and disadvantages to studying plays and theaters.

The research question concerns testing the effects of peer feedback on developing students’ writing performance over time. This study has shown that students in the experimental group performed better at the post-test stage in the overall writing score and its four dimensions after processing writing instructions for 18 weeks. This was in line with the findings of other studies (e.g., [22, 27, 28, 44]), however, no study examines two critical academic writing dimensions (i.e., *task response* and *coherence and cohesion*). The current study extended previous studies and suggested that peer feedback in process writing instructional framework, although not conclusively, can improve medical students’ English writing performance in terms of *task response*, and *coherence and cohesion*.

The improvement in task response for students in the experimental group has suggested that students had realized the importance of presenting relevant and well-supported opinions, and their ability to answer the writing requirements can be improved through the teacher’s instructions on model texts and brainstorming activities in the planning stage. Therefore, they could address the issues with relevant and persuasive statements. This can be supported by the analysis of students’ essays. For example, in the pre-tests, many students tend to write some sentences that did not have a direct relationship with the writing topic such as *“As the medical science continues to develop, people have no choice but to learn new knowledge…”* and *“With the development of the Internet, a wide range of amusements have filled our life”*. Although these sentences had no problems concerning grammatical accuracy, it can be easily recognized that these sentences do not have direct relations with the writing topic *the importance of reading ability, or the way to improve it*. In contrast, in the post-tests, this phenomenon did not occur in most students in the experimental group.

After receiving feedback from peers, students in the experimental group improved in the *lexical resource*, which echoes the findings of several recent studies (e.g., [9, 19, 28]). For example, Jalalzai et al.’s [9] study in a Pakistani school demonstrates that applying peer feedback to teaching practices could effectively broaden L2 students’ vocabulary size and develop their vocabulary knowledge. Meanwhile, making significant revisions during the writing process can motivate L2 writers to use more advanced and sophisticated vocabulary in their essays, and this may help them achieve better performance in *lexical resource*, which is corroborated by the results of Muncie’s [23] study. The improvement of *lexical resource* also can be supported by analysis of students writing. In the pre-test, most of the students can use many vocabularies but with some inaccuracy and they do not excel in spelling even for the very common vocabulary. For instance, they wrote* “…magazines and nowels,” “Currently, investigations on…”*. In the post-test, this group of students can use a more advanced academic vocabulary to express their ideas with rare errors.

Students in the experimental group also made great achievements in *coherence and cohesion* after the peer feedback intervention. Compared with teacher feedback, a significant benefit of peer feedback is that it put great emphasis on providing feedback at the meaning level [30, 38]. That is to say, when students are offering feedback on their peers’ work, they are likely to recognize the inappropriate or incorrect semantic and logical relationships between each statement, which could improve students’ English writing regarding *coherence and cohesion*. Based on the analysis of students’ written texts in the experimental group, one of the most apparent changes happened in the use of conjunctions. In the pre-tests, we found that some students might not have realized the necessity to use conjunctions to organize their sentences logically. Consequently, students wrote sentences like *“I am keen on the ancient history regarding China, it offers me the opportunities to enjoy ancient cultures.”* and *“Practice makes perfect, you should read authentic books regularly.”*. However, our analysis of students’ writing samples in the post-tests has shown that a wide range of conjunctions like “because”, “so” “since” “while” and “but” were accurately used to organize their sentences. This may be because they have understood the logical relationship (e.g., causal relation, adversative relation, and progressive relation) between simple sentences and were able to use specific conjunctions to present their ideas.

The experimental group achieved great progress in *grammatical range and accuracy*, which confirms the empirical findings that peer feedback could significantly develop the grammatical accuracy of learners’ English writing (e.g., [19, 33]). Further, Sato and Lyster [32] attributed this improvement to the fact that grammatical accuracy is an important focus of most students while offering peer back to their peers. Besides, students were able to collaborate with their peers during the provision of feedback, which would give students a chance to exchange information, including grammatical knowledge, with others [17, 20, 28]. At this point, students could also provide their peers with detailed and specific suggestions for improvement according to their actual writing issues so that they could grape the relevant grammatical knowledge effectively. The improvement in *grammatical range and accuracy* could be supported by the analysis of the students’ writings. In the pre-test, various kinds of errors such as lack of subject, improper word order, subject-verb disagreement, and misuse of tense and voice were identified in students’ essays frequently. After the intervention, however, many of them can avoid these errors with the awareness of checking their writing while writing drafts.

Further, students in the experimental group outperformed those in the control group in *task response*, *coherence and cohesion*, *lexical resource*, *grammatical range and accuracy*, and their overall writing scores after 18-week process writing instructions. By using the product-orientated approach, writing teachers in the control group primarily focused on the grammatical accuracy and the vocabulary breath that students used in their compositions and asked students to imitate the writing patterns presented by the model texts in the coursebook without any explanation about the reason and logic behind it. However, it was extremely challenging for students to truly understand the underlying writing patterns of these model texts and thus they were less likely to incorporate the writing patterns into their essays, which is also indicated by Kadmiry [11]. Nevertheless, students in the experimental group used the rubric to give detailed feedback to their peers, which required students to engage in various communicative and interactive teaching activities such as group discussion and brainstorming. This offered students the opportunity to have in-depth communication and collaborate with their peers [16, 17, 20, 21]. Furthermore, during the process of providing and receiving peer feedback, students learned from other’s viewpoints and obtained audience awareness [8, 17, 20, 31], which helped them understand how to present their views and improve the overall quality of the next writing. Besides, students took peer feedback into the recursive process of writing from the first draft to the third draft (i.e., the final draft), which helped them produce better essays from all aspects of writing across drafts and over time.

Several limitations and suggestions for future studies can be proposed. First, the current study was carried out at a single university in China, further research should be conducted to verify the generalizability of the findings in other L2 learning contexts. Second, learner characteristics such as gender, peer feedback literacy, and epistemic beliefs may influence the provision, reception, and engagement of peer feedback (see [3, 26, 27, 45]), while these factors were not included in the current study. Therefore, future studies could take these factors into account so as to reach more a holistic conclusion. Third, in addition to peer feedback, other factors (e.g., students’ academic self-efficacy toward writing and class engagement) may be related to medical students’ L2 academic writing performance. Therefore, more comprehensive investigations should be conducted in the future.

To conclude, this longitudinal interventional study examined the effects of peer feedback on Chinese medical students’ English writing performance over time. Compared with prior studies using the conventional product-oriented writing approach, the finding of the current study suggests that incorporating peer feedback into L2 writing teaching can effectively enhance medical students’ overall writing performance and performance in various aspects, including task response, coherence and cohesion, lexical resource, and grammatical range and accuracy.

## Data Availability

We confirm that the data supporting the findings are available within this article. Raw data supporting this study’s findings are available from the corresponding authors, upon reasonable request.
